# Simultaneous Selective Dorsal Rhizotomy and Baclofen Pump Removal Improve Ambulation in Patients with Spastic Cerebral Palsy

**DOI:** 10.7759/cureus.2791

**Published:** 2018-06-12

**Authors:** TS Park, Brandon A Miller, Junsang Cho

**Affiliations:** 1 Neurological Surgery, Washington University School of Medicine, St. Louis Children's Hospital, St. Louis, USA

**Keywords:** cerebral palsy, intrathecal baclofen pump, selective dorsal rhizotomy, spasticity, neurosurgery

## Abstract

Background: Intrathecal baclofen (ITB) administration via an implanted programmable pump and selective dorsal rhizotomy (SDR) are both used for the treatment of cerebral palsy (CP) spasticity.

Objective: To examine whether SDR can improve ambulation in children who have been receiving ITB therapy for spastic cerebral palsy.

Methods: We reviewed 13 patients who received prior ITB placement with subsequent simultaneous SDR and ITB removal. Patients also completed a follow-up survey to document long-term motor function.

Results: In our 13-patient cohort, patients received ITB treatment for an average of 4.4 $\pm$ 1.8 years and the mean age of ITB removal/SDR was 12.5 $\pm$ 5.8 years. The follow-up period ranged from 3 to 19 months (mean duration: 6.9 $\pm$ 5 months). Pre-operatively, all patients had Gross Motor Function Classification System (GMFCS) scores between 2 and 4. Nine patients were diagnosed with spastic diplegia, two had spastic triplegia and two had spastic quadriplegia. SDR and ITB removal led to improved lower limb spasticity and ambulation. GMFCS scores remained stable in all patients. One patient developed a cerebrospinal fluid (CSF) collection in the abdominal wall due to a CSF leak from the baclofen pump site. All 11 patients who completed the follow-up survey noted improved motor function.

Conclusion: This study demonstrates that SDR can reduce spasticity and improve mobility after years of ITB treatment for spastic cerebral palsy.

## Introduction

Many children with spastic cerebral palsy (CP) receive intrathecal baclofen (ITB) treatment; however, selective dorsal rhizotomy (SDR) is a newer treatment plan with great promise. The primary goal of SDR is the removal of spasticity and the improvement of ambulation. While ITB and SDR are both efficacious, there has been no direct comparison of the two treatments. In fact, there are no reports on the use of the two techniques in combination or sequentially. As a result, patients and families are under the impression that SDR is not an option when ITB fails to reduce spasticity and improves motor functions.

Over the past 11 years, we have treated patients with spastic diplegia and quadriplegia who underwent prior ITB therapy with SDR and simultaneous baclofen pump removal. Here, we report the outcomes for patients receiving ITB and SDR sequentially.

## Materials and methods

This retrospective study was approved by the Institutional Review Board of Washington University School of Medicine (approval # 201605128). We reviewed 13 patients who underwent SDR and baclofen pump removal at the St. Louis Children’s Hospital. Data points, including patient’s age, date of surgery, pre- and post-SDR ambulation, Gross Motor Function Classification System (GMFCS) [1], Modified Ashworth Score (MAS), and medications received, were collected and reviewed.

All patients were evaluated by a neurosurgeon and physical therapist before SDR procedures. ITB was weaned off for at least one month before SDR to prevent baclofen withdrawal symptoms after pump removal. SDR was performed via one-level laminectomy at the level of the conus medullaris that allows access to the L1 to S1 dorsal roots. Our surgical technique was described in detail previously [2]. At the time of SDR, the intrathecal portion of the catheter was removed. Patients had epidural catheters placed for postoperative pain management.

After SDR was completed, patients were repositioned for baclofen pump removal. Separate incisions were made on the back and abdomen. The baclofen pump and catheters were removed. Patients remained on bed rest with epidural analgesia for three days. Patients were discharged home on postoperative day five.

For the follow-up survey, our research team identified eligible patients and collected contact information, in the form of emails and mailing addresses, from electronic patient records at the Center of Cerebral Spasticity at St. Louis Children’s Hospital. We contacted patients via email and phone to obtain verbal consent before conducting the survey. We recorded the responses in a password-encrypted database. Our survey asked patients to report motor function and any additional treatments.

## Results

Thirteen patients underwent baclofen pump removal and simultaneous SDR at a mean age of 12.5 years with a standard deviation of 5.8 years (Table 1). Among the 13 patients, nine were diagnosed with spastic diplegia, two with spastic triplegia, and two with spastic quadriplegia. The average time from ITB initiation to removal was four years (Table 1). After the patients were accepted for SDR, their neurologists gradually weaned ITB therapy in preparation for SDR and ITB pump removal. The patients received no intrathecal baclofen for at least one month before SDR. We were unable to obtain when patient 11 first underwent ITB placement because she was adopted as a child.

**Table 1:** Patient Characteristics

Patient Number	CP Diagnosis	Years w ITB	SDR / ITB Removal Age (years)
1	Diplegia	3	8
2	Diplegia	2	12
3	Diplegia	7	5
4	Diplegia	4	8
5	Diplegia	6	10
6	Triplegia	2	10
7	Diplegia	2	11
8	Triplegia	6	10
9	Quadriplegia	4	9
10	Diplegia	5	22
11	Diplegia	Unknown	23
12	Quadriplegia	5	21
13	Diplegia	7	14
Mean + STDEV		4.4 + 1.8	12.5 + 5.8

The average length of follow-up after SDR was 6.9 months with a standard deviation of five months (Table 2). Of the 13 patients, 12 maintained the same GMFCS level and one patient improved from a score of 4 to 3. Seven patients improved their ambulation with SDR/ITB removal, and six patients' ambulation was unchanged. Additionally, all patients experienced a reduction of spasticity, progressing from a mean modified lower limb Ashworth score of 2.3 $\pm$ 0.5 to 0.007 $\pm$ 0.03 at their postoperative assessment (Table 2). All patients resolved ankle clonus and hyperreflexia after SDR, indicating a reduction in spasticity for all patients post-SDR.

**Table 2:** Outcomes of Simultaneous Removal of Baclofen Pump and Selective Dorsal Rhizotomy

Patient Number	Preop GMFCS	Postop GMFCS	Ambulation Change with SDR / ITB Removal	Pre-SDR MAS	Pre-SDR Ankle Clonus and Hyperreflexia	Post-SDR Ankle Clonus and Hyperreflexia	Post-SDR/ITB Removal MAS	Follow-Up (Months)
1	3	3	Improved	2.8	Yes	No	0	3.3
2	2	2	Same	1.8	Yes	No	0.1	4.6
3	3	3	Same	2.3	Yes	No	0	4.6
4	3	3	Improved	2.4	Yes	No	0	16.7
5	3	3	Same	2.1	Yes	No	0	4.6
6	3	3	Improved	2.8	Yes	No	0	6.2
7	3	3	Same	2.5	Yes	No	0	19.3
8	2	2	Improved	2.3	Yes	No	0	5.0
9	3	3	Same	3.3	Yes	No	0	4.1
10	2	2	Same	2.0	Yes	No	0	6.7
11	3	3	Improved	2.5	Yes	No	0	4.1
12	3	3	Improved	1.8	Yes	No	0	4.3
13	4	3	Improved	1.7	Yes	No	0	6.8
Mean + STDEV	2.8 + .55	2.8 + .54		2.3 + ​​​​​​​.46			.007 + ​​​​​​​0.03	6.9 + ​​​​​5

Patients were also assessed with a follow-up survey that addressed motor function. We asked patients how their mobility has changed since surgical intervention with respect to five measures: walking ability, sitting posture, standing ability, transitional movements, and balance while walking. Two patients were lost to follow-up. The patient responses were overwhelmingly positive, as none of them reported worsened conditions since SDR.

Regarding walking, seven out of 11 patients reported marked improvement, two improved somewhat, and only two patients reported no changes (Table 3). Concerning sitting, six out of 11 patients had marked improvement, three improved somewhat, and only two had no changes. Regarding standing ability, four patients improved markedly and seven patients somewhat improved. Regarding transitional movements, six patients reported significant improvement, four patients reported somewhat improved movement, and only one patient experienced no changes. Lastly, concerning balance while walking, four patients had remarkable improvements, six patients improved somewhat, and one patient had no change.

**Table 3:** Details of Patient Response to Survey

Patient Number	Change in “Walking”	Change in “Sitting Posture”	Change in “Standing”	Change in “Transition Movements”	Change in “Balance While Walking”
1	Improved (Markedly)	Improved (Markedly)	Improved (Somewhat)	Improved (Markedly)	Improved (Markedly)
2	Improved (Markedly)	Improved (Markedly)	Improved (Somewhat)	Improved (Somewhat)	Improved (Markedly)
3	No response to survey				
4	No Change	Improved (Somewhat)	Improved (Somewhat)	Improved (Somewhat)	Improved (Somewhat)
5	No response to survey				
6	Improved (Markedly)	Improved (Somewhat)	Improved (Somewhat)	Improved (Somewhat)	Improved (Somewhat)
7	Improved (Markedly)	Improved (Markedly)	Improved (Markedly)	Improved (Markedly)	Improved (Somewhat)
8	Improved (Somewhat)	Improved (Markedly)	Improved (Somewhat)	Improved (Markedly)	Improved (Somewhat)
9	Improved (Somewhat)	Improved (Markedly)	Improved (Somewhat)	Improved (Somewhat)	Improved (Somewhat)
10	Improved (Markedly)	No Change	Improved (Markedly)	No Change	Improved (Markedly)
11	Improved (Markedly)	Improved (Markedly)	Improved (Markedly)	Improved (Markedly)	No Change
12	No Change	No Change	Improved (Somewhat)	Improved (Markedly)	Improved (Somewhat)
13	Improved (Markedly)	Improved (Somewhat)	Improved (Markedly)	Improved (Markedly)	Improved (Markedly)

Case report 

Patient 7

This 11-year-old boy was born prematurely and diagnosed with spastic diplegia at one year of age. He used a walker to ambulate at four years of age and started ITB therapy at nine years of age. Before the ITB therapy, he could walk with a walker in all environments. During the ITB therapy, he became weaker as evidenced by the use of a wheelchair in most environments. On the pre-SDR examination, he showed severe hamstring and heel cord contractures in addition to hyperreflexia and ankle clonus. He underwent simultaneous SDR and removal of a baclofen pump. Four months after SDR, he received percutaneous lengthening of the hamstrings and heel cords. Video 1 shows this patient’s ambulatory improvement from pre-operation to post-operation.

**Video 1:** Patient 7

Patient 13

This male adolescent suffered bacterial meningitis in infancy followed by severe motor impairment. He underwent baclofen pump placement at two years of age; subsequently, he experienced improvements in ambulation. He underwent one revision of the baclofen pump. At 14 years of age, he walked with a walker in a protected environments and his pump started to deteriorate. As a result, after consultation with St. Louis Children’s Hospital, the family chose to undergo the simultaneous removal of the baclofen pump and SDR. On the pre-SDR examination, he showed hyperreflexia, ankle clonus, and hamstring contractures. After undergoing hamstring lengthening orthopedic surgery three months following SDR, he greatly improved and currently walks in all environments. Video 2 shows the patient’s improvement from pre-surgical intervention to post-intervention.

**Video 2:** Patient 13

## Discussion

We report the option of SDR for patients who receive long-term ITB therapy for spastic CP but exhibit deteriorating mobility despite the treatment. We discovered that patients with GMFCS levels ll and lll suffered declining ambulatory function during long-term ITB therapy, and the combination of SDR/termination of ITB therapy resulted in qualitative improvements in motor function.

The literature contains a single report of outcomes after the combined treatment of SDR and the removal of the ITB pump. Ingale et al. found in 10 non-ambulatory spastic CP patients (GMFCS levels lV and V) that SDR was more effective than ITB in reducing spasticity and daily care. In addition, they found that SDR was a cheaper treatment option than ITB therapy. They did not present details of functional changes in their series [3].

Spasticity for CP patients contributes to the development of well-documented early aging manifested as muscle weakness, decreased endurance, and muscle and joint pain [4-5]. In our 30-year experience with more than 3,800 patients treated with SDR for spastic CP, we also found that early aging occurs even in mild spastic diplegia and elimination of spasticity is required to reverse or prevent the pathologic process. It is pertinent that our patients showed persistent spasticity and the concomitant decline of the ambulation during the ITB therapy, and SDR improved the quality of ambulation postoperatively. The clinical course indicates that the persistent spasticity during ITB therapy can cause the decline of ambulatory function. Other authors have also reported that intrathecal baclofen in animals and epidural baclofen in children caused transient motor weakness [6-7]. Whether prolonged ITB decreases patients' motor strength requires further investigation.

It is worthwhile to mention a previous report that compared SDR with ITB therapy in children with spastic CP. Kan et al. compared 71 children who underwent SDR for the treatment of spasticity with 71 children who underwent ITB pump placement. The two groups had comparable age and preoperative GMFCS levels. Both procedures resulted in a high degree of patient satisfaction. Compared with ITB therapy, SDR produced greater improvement in lower-extremity tone, passive range of movements, and gross motor function. In addition, fewer patients in the SDR group required subsequent orthopedic procedures [8].

## Conclusions

Our results support that SDR can be an option for ambulatory patients with spastic CP who fail to improve with prolonged ITB therapy. Long-standing ITB therapy does not preclude the use of SDR to treat spasticity and improve the motor functions.
